# *Ceraphron krogmanni* (Hymenoptera: Ceraphronidae), a new species from Lower Saxony with unusual male genitalia

**DOI:** 10.3897/BDJ.6.e24173

**Published:** 2018-04-17

**Authors:** Jonah M Ulmer, Istvan Miko, Andrew R Deans

**Affiliations:** 1 Pennsylvania State University, University Park, United States of America; 2 Frost Entomological Museum, Penn State University, University Park, United States of America

**Keywords:** *
Ceraphron
*, morphology, taxonomy, genitalia

## Abstract

**Background:**

Male genitalia phenotypes of *Ceraphron* (Jurine, 1807) are informative for species delimitation, but due to their minute size, these characters have not been used extensively. Recent developments in visualisation techniques, *e.g.* confocal laser scanning microscopy and high resolution bright field imaging, allow for more thorough examination of these minute anatomical structures and the development of a robust, male genitalia-based taxonomic system. We also establish a character set, a template, that will facilitate future revisions of these wasps.

**New information:**

*Ceraphron krogmanni* sp. nov. is described with outsized male genitalia and multiple diagnostic traits that are unique amongst *Ceraphron* species.

## Introduction

The family Ceraphronidae holds 302 species, classified in 14 genera. More than 90% of the species belong in two genera, *Ceraphron* (n=185) and *Aphanogmus* (n=94) (Johnson and Musetti 2004, Various Contributors 2017). Genus-level identifications of female ceraphronids are sometimes complicated because the only diagnostic feature, the width/height ratio of the mesosoma (higher than long in *Aphanogmus* and wider than high in *Ceraphron*) is dubious (*e.g.* females of *Aphanogmus fumipennis* Thomson 1858 species group are often as wide as high, Mikó, *pers. obs.*). On the other hand, male *Ceraphron* specimens are easy to distinguish from *Aphanogmus* by the morphology of the antenna; the flagellomeres in *Ceraphron* are cylindrical and covered with sensillum trichodeum curvatum (sickle-shaped sensilla), while, in *Aphanogmus*, they are trapezoidal in lateral view and equipped with erect and elongate setae Mikó and Deans (2009).

The external morphology of Ceraphronoidea is monotonous relative to other microhymenoptera (*e.g.* other taxa in Proctotrupomorpha; Klopfstein et al. 2013) and the few characters that do appear suitable for species delineation (*e.g.* anatomical line ratios or microsculpture) are often affected by allometry (Mikó and Deans 2012, Mikó et al. 2016). On the other hand, male genitalia characters have revealed themselves to be consistently informative systematically (Dessart 1972, Dessart 2001, Mikó and Deans 2012). Studying ceraphronid male genitalia is difficult with the traditional taxonomist tool set, due to the structures' extremely small size (100 - 200µm) and a functional, male genitalia-based taxonomic system remaining elusive. These limitations have restrained further exploration of ceraphronid fauna and only species with very peculiar external traits have been described (Dessart 1984, Dessart 1975, Dessart 1981). The male genitalia have been documented for only 13 species of *Ceraphron* (Dessart 1963, Dessart 1964, Dessart 1965, Dessart 1975,Dessart 1981, Dessart 1989).

After decades of dysfunction in *Ceraphron* taxonomy (the last species, *C. bestiola*, was described 22 years ago, based on a single female specimen from Switzerland by Cancemi and Dessart 1995), we describe a new species with distinct male genitalia. We also establish a character set, a template, that will facilitate future revision of these wasps.

## Materials and methods

Specimens were examined and dissected under an Olympus SZX16 stereomicroscope, with an Olympus SDF PL APO 1× PF objective (115×) and an SDF PL APO 2× PFC Objective (230×). Dissections were performed using #2 insect pins and Rubis 5A-SA forceps. Genitalia were removed and placed on to a separate concave slide in glycerol. Diagnostic measurements were performed on the specimens and genitalia using a KR 851 stage micrometer attached to the examination stereomicroscope.

Bright field images were taken with an Olympus ZX41 compound microscope and attached Olympus DP71 digital camera. Genitalia were imaged in glycerol and specimens, including dissected segments, were imaged on Bostik Blu-Tack Reusable Adhesive (Ellsworth Adhesives, Germantown, WI, USA) to stabilise and position for imaging. Images were aligned using Zerene Stacker Version 1.04 Build T201404082055. Annotation and colour correction was performed in Adobe Photoshop CS4.

Genitalia were imaged using an Olympus FV10i Confocal Laser Scanning Microscope (CLSM) following the protocol from Mikó et al. (2014) using 60× objective.

Specimen data, including figures and character states were imported to MX (http://mx.phenomix.org). The diagnostic characters, description and materials examined were autogenerated by the same content management system. Morphological terms used in the description and diagnosis are derived from phenotype class based ontologies.

All phenotypic descriptions were expressed as semantic statements using Protégé Version 5.0.0 (Build beta 17) using the method provided by Balhoff et al. (2013) and Mikó et al. (2014).

The holotype is deposited at the State Museum of Natural History (SMNS) in Stuttgart, Germany and the paratype will be retained at the Pennsylvania State University Frost Entomological Museum (PSUC).

## Taxon treatments

### Ceraphron krogmanni sp. n.

urn:lsid:zoobank.org:act:E762860F-DBB7-47AE-A94B-543290842491

#### Materials

- **Type status:** Holotype. **Occurrence:** catalogNumber: PSUC_FEM 86417; recordedBy: L. Krogmann, R. Peters; individualCount: 1; sex: male; lifeStage: adult; preparations: glycerol; occurrenceID: urn:uuid:675a5573-994c-4136-be14-03ab9de2aace; **Taxon:** scientificName: Ceraphron krogmanni; kingdom: Animalia; phylum: Arthropoda; class: Insecta; order: Hymenoptera; family: Ceraphronidae; genus: Ceraphron; specificEpithet: krogmanni; taxonRank: species; **Location:** locationID: 91c31a2d21f8ea1df5681e7f945ec536; continent: Europe; country: Germany; locality: Niedersachen, Lkr. Lüchow-Dannenberg, Pevestorf, Deichvorland & Deich; verbatimCoordinates: N53°03'49", E11°28'27"; decimalLatitude: 53.063611; decimalLongitude: 119.4338; georeferenceProtocol: label; **Identification:** identifiedBy: Jonah M. Ulmer; dateIdentified: 2016; **Event:** samplingProtocol: sweeping; eventDate: 08/06/2013; **Record Level:** language: en; institutionID: SMNS; collectionID: urn:lsid:biocol.org:col:34840; institutionCode: State Museum of Natural History (SMNS); collectionCode: SMNS_Hym_T00665; basisOfRecord: PreservedSpecimen
- **Type status:** Paratype. **Occurrence:** catalogNumber: PSUC_FEM 86221; recordedBy: L. Krogmann, R. Peters; individualCount: 1; sex: male; lifeStage: adult; preparations: glycerol; occurrenceID: urn:uuid:675a5573-994c-4136-be14-03ab9de2aace; **Taxon:** scientificName: Ceraphron krogmanni; kingdom: Animalia; phylum: Arthropoda; class: Insecta; order: Hymenoptera; family: Ceraphronidae; genus: Ceraphron; specificEpithet: krogmanni; taxonRank: species; **Location:** locationID: 91c31a2d21f8ea1df5681e7f945ec536; continent: Europe; country: Germany; locality: Niedersachen, Lkr. Lüchow-Dannenberg, Pevestorf, Deichvorland & Deich; verbatimCoordinates: N53°03'49", E11°28'27"; decimalLatitude: 53.063611; decimalLongitude: 119.4338; georeferenceProtocol: label; **Identification:** identifiedBy: Jonah M. Ulmer; dateIdentified: 2016; **Event:** samplingProtocol: sweeping; eventDate: 08/06/2013; **Record Level:** language: en; institutionID: PSUC; collectionID: http://grbio.org/cool/29fv-ztxs; institutionCode: Frost Entomological Museum (PSUC); collectionCode: Insects; basisOfRecord: PreservedSpecimen

#### Description

Body length universal: 0.9—1.1 mm.

##### Coloration

Colour hue pattern female: NOT CODED. Colour intensity pattern female: NOT CODED. Colour hue pattern male: brownish, legs yellowish, fore wing brown, with a transverse discoloured band at level of stigmal vein. Colour intensity pattern male: flagellum, tibiae and tarsi lighter than scape, pedicel, mandible, tegula, coxae and femora.

##### Head

Foveolate sculpture on body count: present on mesosoma and frons. Head width vs. head height: HW:HH=0.9—1.0. Head width vs. interorbital space (HW/IOS) Female: NOT CODED. Head width vs. interorbital space (HW/IOS) Male: 1.4—1.5. Head width vs. head length lateral view (HW / HL): 1.6. Maximum eye diameter vs. minimum eye diameter: 1.1—1.2. Dorsal carina of occipital depression presence: absent. Dorsal carina of occipital depression medial continuity: NOT CODED. Occipital carina sculpture: smooth (Fig. 3a). Median flange of occipital carina count: present. Submedial flange of occipital carina count: present. Dorsal margin of occipital carina vs. dorsal margin of lateral ocellus in lateral view: occipital carina is ventral to lateral ocellus in lateral view. Preoccipital lunula count: present. Preoccipital ridge presence: present. Preoccipital furrow count: present. Preoccipital furrow anterior extension: adjacent anteriorly to the posterior margin of the median ocellus. Preoccipital furrow anterior region vs. posterior region sculpture: crenulate in its entire length. Preoccipital furrow anterior region width vs. posterior region width: as wide anteriorly as posteriorly. Preoccipital carina count: present. Preoccipital carina shape: complete. Preoccipital carina and occipital carina structure: occipital carina complete, preoccipital carina fused laterally with preorbital carina. Setal pit on vertex size: smaller than diameter of scutes. Female ocular ocellar line (OOL): posterior ocellar line (POL): lateral ocellar line (LOL): NOT CODED. Male ocular ocellar line (OOL): posterior ocellar line (POL): lateral ocellar line (LOL): 2:1—1.2:1—1.2 (Fig. 1a). postocellar carina count: absent. Preocellar pit count: present. Randomly sized areolae around setal pits on upper face count: absent. Antennal scrobe count: present. Carina delimiting antennal scrobe count: present. Transverse striation on upper face: present. Transverse scutes on upper face count: present. Region on upper face width transverse scutes lateral limit: extending entire width of frons. Transverse frontal carina count: present (Fig. 3b). Frontal ledge count: absent. Rugose region on upper face count: absent. Ocellar fovea margin sharpness: blunt. Anterior ocellar fovea shape: fovea extended ventrally into facial sulcus, reaching dorsal margin of antennal scrobe. Facial pit count: no external corresponding structure present. White, thick setae on upper face count: present. Ventromedian setiferous patch and ventrolateral setiferous patch count: absent. Supraclypeal depression count: present. Supraclypeal depression structure: present medially, inverted U-shaped. Intertorular carina count: present. Median process on intertorular carina presence: absent. Intertorular ridge vs. epistomal ridge: separated. Intertorular area count: present. Median region of intertorular area shape: flat. Torulus position relative to anterior ocellus and distal margin of clypeus: torulus not reaching epistomal sulcus, closer to distal margin of clypeus than anterior ocellus. Torulo-clypeal carina count: present. Subtorular carina count: present. Subantennal groove count: absent. Subantennal groove structure: NOT CODED. Posterolateral process of gena count: present. Ocular impression and post ocular orbital carina count: present. Ocular impression sculpture: scalloped (foveae composing ocellar impression adjacent, sometimes not separated from each other). Median conjunctiva of cardines count (median fusion of left and right cardines): absent (cardines are fused). Maxillary palpomeres count: 4. Mandibular tooth count: 2. Mandibular lancea count: NOT CODED.

##### Mesosoma and Metasoma

Mesosoma shape: not compressed laterally, as wide as high or wider than high (Fig. 1b). Weber length: WL=370—390µm. Pronope count: present. Transverse pronotal sulcus (anterodirsal branch of pronotal y) count: absent. Epomial carina presence: present. Posterodorsal branch of pronotal Y count: absent. Occlusor muscle apodeme for the occlusor muscle of the anterior thoracic spiracle count: NOT CODED. Occlusor muscle apodeme for the anterior thoracic spiracle structure: NOT CODED. Pit corresponding to the apodeme for the occlusor muscle of the anterior thoracic spiracle pit count: NOT CODED. Atrium of anterior mesothoracic spiracle count: NOT CODED. Atrium of the anterior thoracic spiracle size: NOT CODED. Ventrolateral invagination of the pronotum count: present. Annullar pronotum count: present. Ventromedian region of pronotum and anteroventral region of mesopectus continuity: pronotum and mesopectus continuous ventromedially. Lateroventral invagination of the propleuron presence: absent. Mesonotal fossa of the pronotum and pronotal condyle of the mesonotum presence: present. Mesoscutal length vs. anterior mesoscutal width: MscL/AscW=1.5—1.7. Mesonotum anterolateral margin shape: square. Median mesoscutal sulcus count: present. Median mesoscutal sulcus posterior end: adjacent to transscutal articulation. Scutoscutellar sulcus vs. transscutal articulation: adjacent. Notaulus count: present. Anterior mesoscutal width vs. posterior mesoscutal width: AscW/PscW=0.3—0.5. Notaulus posterior end location: anterior to transverse midline of mesoscutum. Posterior end of notaulus vs. posterior end of antero-admedian line: NOT CODED. Transscutal articulation completion: complete. Scutes on posterior region of mesoscutum and dorsal region of mesoscutellum count: present. Scutes on posterior region of mesoscutum and dorsal region of mesoscutellum convexity: flat. Lateral carina on the mesoscutellum presence: present. Axillular carina count: present. Axillular carina shape: left and right carina are separated posteromedially. Axillular setae presence: present. Posterolateral margin of mesoscutellum shape: blunt. Posteromedian process of the mesoscutellum count: present. Posteromedian process of the mesoscutellum shape: blunt. Anteromedian projection of the metanoto-propodeo-metapecto-mesopectal complex count: present. Anteromedian projection of the metathorax-propodeum complex shape: bilobed. Anteromedian projection of the metathorax-propodeum complex curvature lateral in view: straight. Sternaulus count: present. sternaulus length: elongate, exceeding 3/4 of mesopleuron length at level of sternaulus. Longitudinal striae extending from crenulae of anterior mesopleural sulcus to Mesopleural pit count: NOT CODED. speculum ventral limit: NOT CODED. Mesometapleural sulcus count: present. Ventral invagination of mesometapleural sulcus presence: absent. Epicnemial carina count: complete. Epicnemial pit count: present. Epicnemium posterior margin shape: anterior discrimenal pit present; epicnemial carina curved. Mesodiscrimen count: present. Anterior metapleural carina count: absent. Anterior metapleural carina completion: incomplete. Metapleural carina count: present. Metapleural carina vs. propodeal spiracle: metapleural carina extending ventrally of propodeal spiracle. Ventral projection of the metapleural carina presence: absent. Ventral invagination of the metapleural carina: absent. Propodeal spiracle dilator muscle apodeme pit location: posterodorsally of the metapleural carina. lateral propodeal carina count: present. Lateral propodeal carina shape: inverted "V'' (left and right lateral propodeal carinae are adjacent medially at their intersection with antecostal sulcus of the first abdominal tergum). Median propodeal carina count: present. Posterior propodeal projection count: present. Posterior propodeal projection shape: simple. Propodeal and metacoxal verricules presence: absent. Posterodorsal metapleural area shape: NOT CODED. Posterior line of the posterodorsal metapectal area count: absent. Mesofurca vs. metadiscrimenal lamella continuity: fused. Transverse line of the metanotum-propodeum vs. antecostal sulcus of the first abdominal tergum: NOT CODED. Carina limiting posteriorly antecosta count: NOT CODED. Line of separation between the lateral margin of the acrotergite of the first abdominal tergum and the posterodrosal metapleural area count: absent. Posterior margin of nucha in dorsal view shape: straight. Stigmal vein of fore wing count: present. Stigmal vein length vs. pterostigma marginal length: stigmal vein longer than the pterostigma marginal length. Pterostigma of fore wing count: absent (Fig. 5a). Hind wing reduction: well developed. Calcar shape: simple. Mesotibial spur count: 1. Mesobasicoxa width vs. metabasicoxa width: Metabasicoxa distinctly wider than mesobasicoxa. Posterior mesosomal comb count: absent. Anterior mesothoracic spiracle occlusor muscle site of origin: NOT CODED. Pronoto-mesobasalar muscle site origin: NOT CODED. Pronoto-procoxal muscle origin: NOT CODED. Prophragmo-postoccipital muscle site of origin: NOT CODED. Mesonoto-mesotrochanteral muscle presence: NOT CODED. Posterior mesonoto-metanotal muscle site of origin anterior limit: NOT CODED. Second and third mesopleuro-third axillary sclerite of fore wing muscle site of origin: NOT CODED. Mesopleuro-mesocoxal muscle site of origin: NOT CODED. Mesofurco-mesotrochanteral muscle presence: NOT CODED. Mesofurco-mesotrochanteral muscle relative position: NOT CODED. Mesofurco-mesotrochanteral muscle site of insertion: NOT CODED. S1 length vs. shortest width: NOT CODED. Transverse carina of petiole count: present (Fig. 2a). Transverse carina on petiole shape: concave. Basal, longitudinal carinae on syntergum count: more than 5 (Fig. 2b). Transverse sulcus of first metasomal sternum count (S1 count): absent. Waterston's evaporatorium count: present. Waterston's evaporatorium shape medially: not paired, single median evaporatorium present. Acrotergal calyx of Waterston’s evaporatorium count: acrotergal calyx absent.

##### Male Genitalia

Median conjunctiva of male T9 count: absent. Row of short setae delimiting apical, cercus-bearing area of male T9: present. Male T10 shape: folded along median weakly sclerotised line. Median part of male S8 structure: not constricted medially, distal margin concave. Anterior margin shape of male S9: concave. Proximal margin part of male S9 shape: concave. Male S9 distal setal line / setal patch count: distal setae composing transverse setiferous line(s). Distomedian, hairless area (interrupting transverse row of setae or patch) on abdominal sternum 9 count: present (distal setiferous patch / line separated medially). Distal margin of male S9 shape: straight. Proximolateral corner of male S9 shape: acute. proximal lobe of vas deferens: NOT CODED. Distodorsal margin of cupula shape: straight (Fig. 4a). Cupula length vs. gonostyle-volsella complex length: cupula less than 1/2 the length of gonostyle-volsella complex in lateral view. Proximodorsal notch of cupula count: absent (Fig. 4a, b). Proximodorsal notch of cupula shape: NOT CODED. proximodorsal notch of cupula width vs. length: NOT CODED. Ventral part of cupula shape: cupula ventromedially is not extended more proximally than dorsomedially. Dorsal submedian impression of cupula count: present. Distoventral submedian corner of the cupula count: absent. Proximodorsal apodeme of cupula count: present. Proximolateral projection of the cupula shape: NOT CODED. Harpe presence: present. Harpe length: harpe shorter than gonostipes in lateral view. Lateral setae of harpe count: present. Lateral margin of harpe shape: widest point of harpe is at its articulation site with gonostyle-volsella complex (Fig. 4c, d). Sensillar ring area of harpe orientation: medially. Distodorsal setae of sensillar ring of harpe length vs. harpe width in lateral view: NOT CODED. Distoventral seta bearing projection of the harpe count: present. Distodorsal setae of sensillar ring of harpe orientation: NOT CODED. Proximomedial brush of the harpe count: present. Distal margin of harpe in lateral view: straight. Proximomedial apodeme of harpe count: absent. Lateral setae of harpe orientation: oriented distally. Proximodorsal projection of harpe accommodating the gonossiculus count: absent. Proximoventral margin of gonostyle/volsella complex shape: convex, pointed proximally. Distal end of dorsomedian conjunctiva of the gonostyle-volsella complex shape: NOT CODED. Dorsomedian conjunctiva of the gonostyle-volsella complex count: absent. Dorsomedian conjunctiva of the gonostyle-volsella complex length relative to length of gonostyle-volsella complex: NOT CODED. Gonostyle-volsella complex proximoventrally continuity: discontinuous, ventromedian conjunctiva of gonostyle-volsella complex complete, reaching proximal margin of gonostyle-volsella complex. Proximodorsal inflection of cupulal margin count: present. Gonostyle/volsella complex dorsally continuity: discontinuous, dorsomedian conjunctiva of gonostyle complete, extending between proximal and distal margins of gonostyle. Gonostyle/volsella complex proximodorsal margin shape: straight or slightly concave. Medioventral area of gonostyle/volsella complex orientation: horizontal. Distodorsal submedian notch of gonostyle/volsella complex count: absent. Submedian conjunctiva on distoventral margin of gonostyle/volsella complex: length (range of fusion of parossiculus/parossiculus complex from gonostipes): NOT CODED. Medioventral conjunctiva of the gonostyle-volsella complex presence (fusion of parossiculi): medioventral conjunctiva present (parossiculi independent or fused proximally). Parossiculus count (parossiculus and gonostipes fusion): absent (fused with the gonostipes). Apical parossiculal seta number: one. Distal projection of the parossiculus count: absent. Digital teeth orientation: dorsally. Penisvalva proximal region curvature: curved dorsally. Dorsal apodeme of penisvalva count: NOT CODED. Distal projection of the penisvalva count: absent. Ventromedian apodeme of aedeagus count: absent. Distal part of aedeagus and gonostyle continuity: continuous. Sensillar plate of the aedeagus shape: distinctly less than half as wide as the male genitalia. Mediolateral S9-cupulal muscle site of origin: laterally from anterolateral corner of S9. Lateral S9-cupulal muscle site of insertion: medially on the ventral part of cupula. Lateral S9-cupulal muscle subdivision: NOT CODED. Dorsomedial cupulo-gonostipal muscles orientation: converging proximally. Dorsolateral cupulo-gonostipal muscle count: present. Ventrolateral cupulo-gonostipal muscle count: present. Ventromedial cupulo-gonostyle muscle count: absent. Ventral gonostipo-penisvalval muscles site of origin—proximal extension: not extending distally on parossiculus. Lateral gonostyle-penisvalva muscle count: absent. Penisvalvo-gonossiculal muscle count: NOT CODED. Gonostipo-parossiculal muscle count: NOT CODED. Gonostyle/volsella complex-volsella muscle site of insertion: NOT CODED. Lateral intrinsic muscle of volsella: site of insertion: NOT CODED. Medial intrinsic muscle of the volsella count: NOT CODED. Parossiculo-penisvalval muscle count: NOT CODED. Proximal gonostipo-harpal muscle count: NOT CODED. Distal gonostipo-harpal muscle count: NOT CODED. Proximal gonostipo-harpal muscle site of origin: NOT CODED. Distal gonostipo-harpal muscle site of origin: NOT CODED. Distal gonostipo-harpal muscle gonostipal site of origin: NOT CODED.

##### Antennae

Flagellar scrobe of the scape count: absent. Male flagellomeres shape: cylindric. 6th male flagellomere length vs. width, "sensillar" view: short, 1—1.4 times as long as wide. Length of setae on male flagellomere vs. male flagellomere width: setae shorter than width of flagellomeres. Male F1 length vs. pedicel length: 2.5—3.0. Male scape length vs. combined length of F1+F2: longer or equal. Whorled rows of erect, elongate setae on male flagellomeres count: absent. Male F6 length vs. combined length of F7+F8: Shorter than length of flagellomere 7+8. Male F1 length vs. male F2 length: 1.2—1.4. Male scape length vs. pedicel length: 4.1—4.3. Number of flagellomeres with male specific ventral sensilla: F5—9. Multiporous plates on male flagellomeres count: present. Male flagellomere branches count: None (Fig. 5b). Branch of male F5 length vs. length of male F5: NOT CODED. Branch of male flagellomere 5 length compared to flagellomere 6: NOT CODED. Basal resilin-rich area of male antennal branches count: NOT CODED. Female F1 length vs. pedicel length: NOT CODED. Female 9th flagellomere length: NOT CODED.

#### Diagnosis

Based on the presence of sickle-shaped sensilla on the male flagellomeres and the dorsoventrally compressed mesosoma (wider in dorsal view and high in lateral view), the new species belongs to the genus *Ceraphron*. *Ceraphron krogmanni* differs from all other *Ceraphron* species by the presence of a proximomedial harpal brush (mhb:Fig. 4c, d), the presence of ventromedial and dorsomedial ridges of the basal ring (vmr, dmr:Fig. 4a, b) and the unusually large male genitalia Fig. 2b (more than half the length of the syntergum in *C. krogmanni* whereas less than 1/3 of the syntergum in other *Ceraphron* species).

#### Etymology

The species epithet is a patronym, honouring Lars Krogmann, Staatliches Museum für Naturkunde Stuttgart, who collected the specimens observed in this study.

## Supplementary Material

XML Treatment for Ceraphron krogmanni

## Figures and Tables

**Figure 1a.**
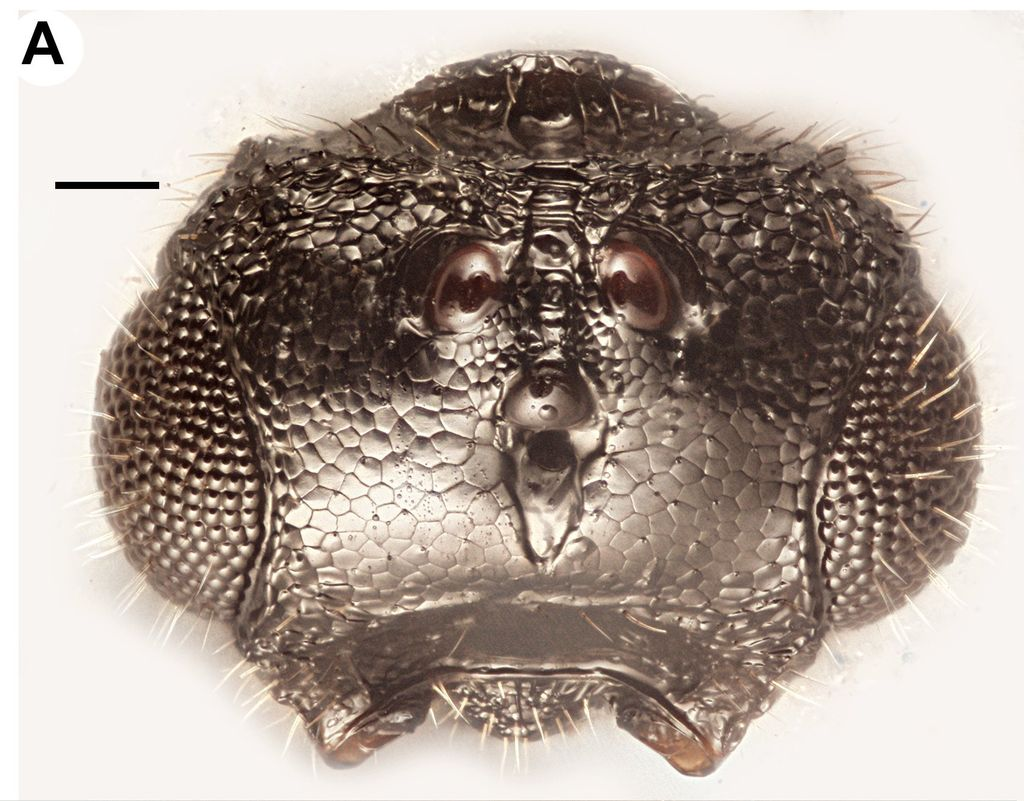
Head in dorsal view

**Figure 1b.**
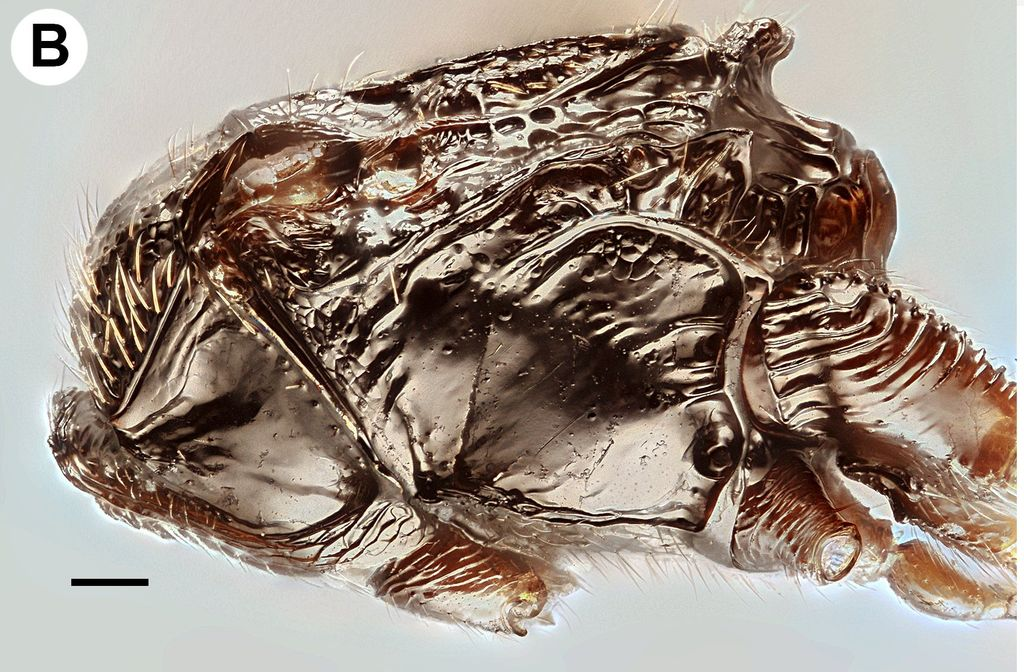
Mesosoma in lateral view

**Figure 2a.**
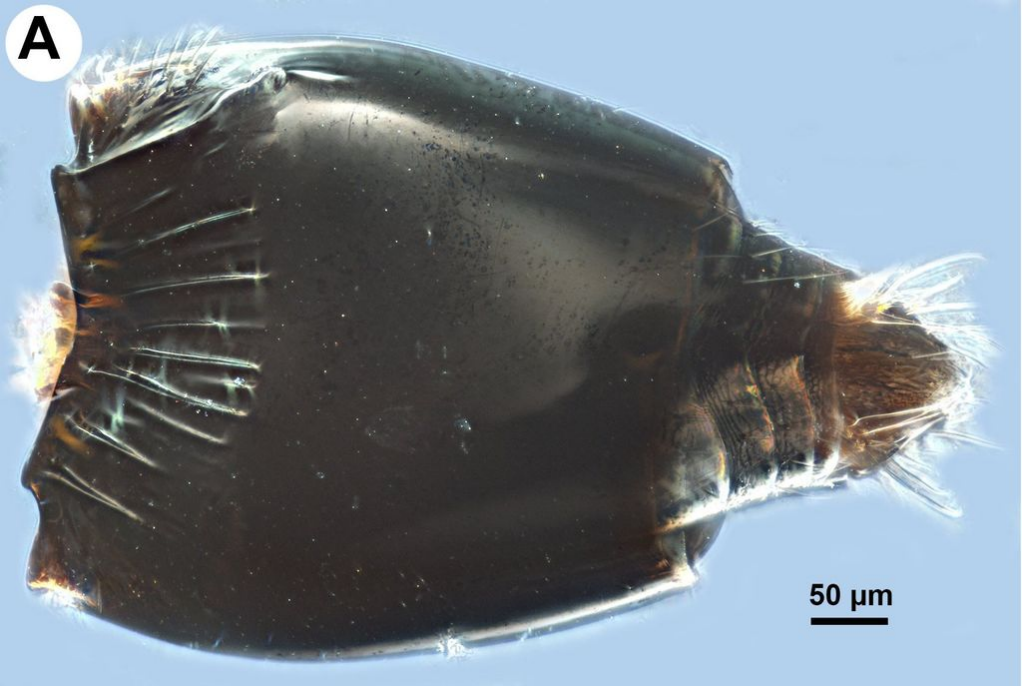
Metasome in dorsal view.

**Figure 2b.**
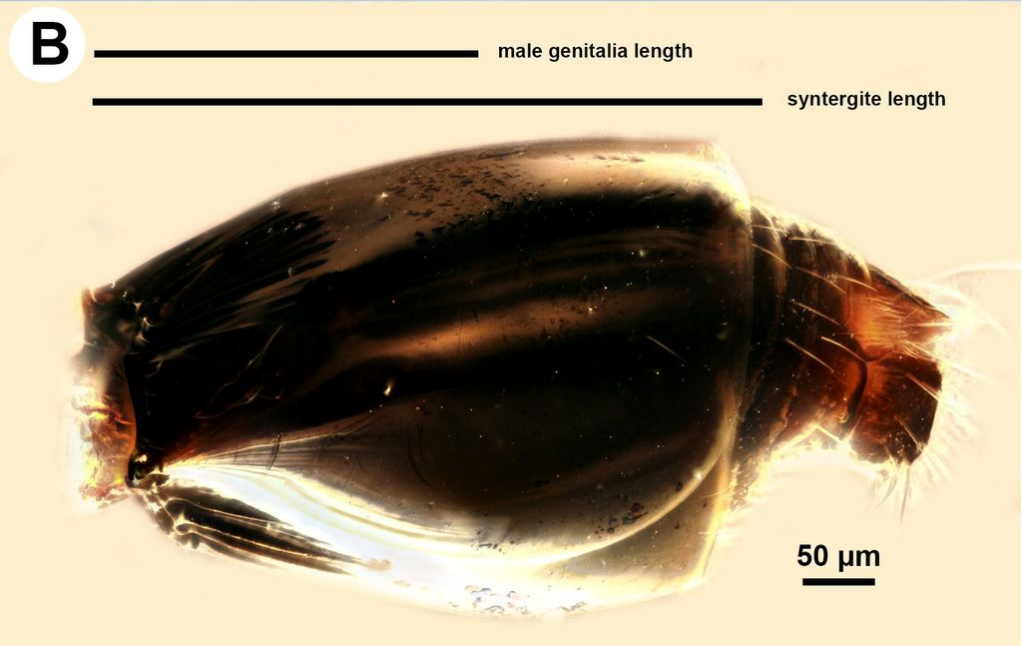
Metasoma in lateral view showing length of genitalia in relation to syntergite.

**Figure 3a.**
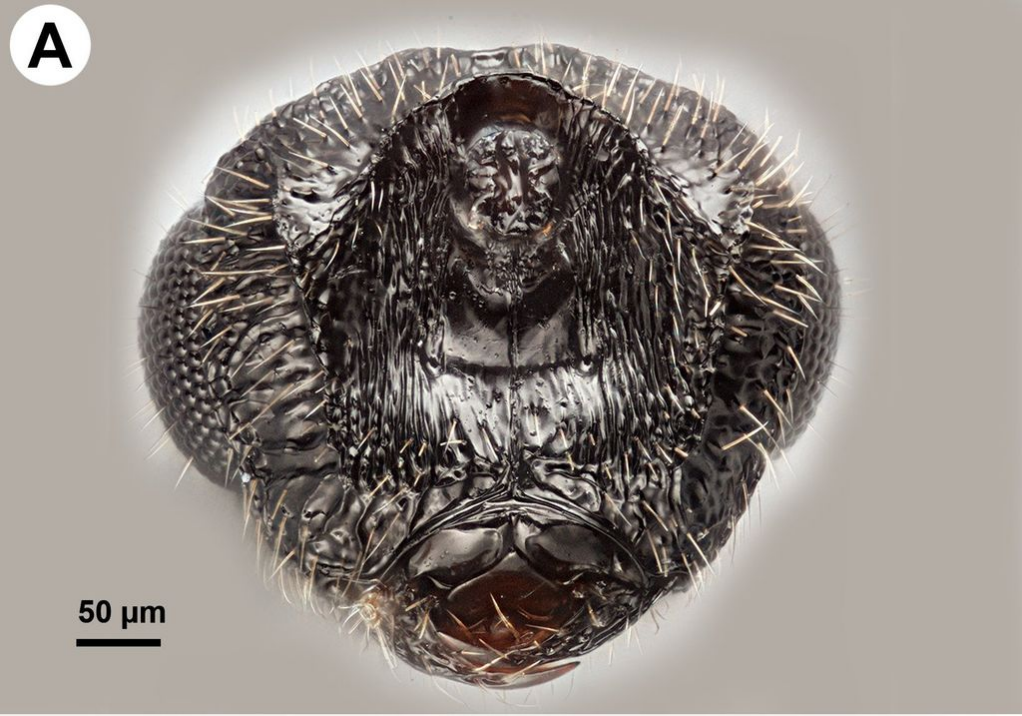
Head in posterior view.

**Figure 3b.**
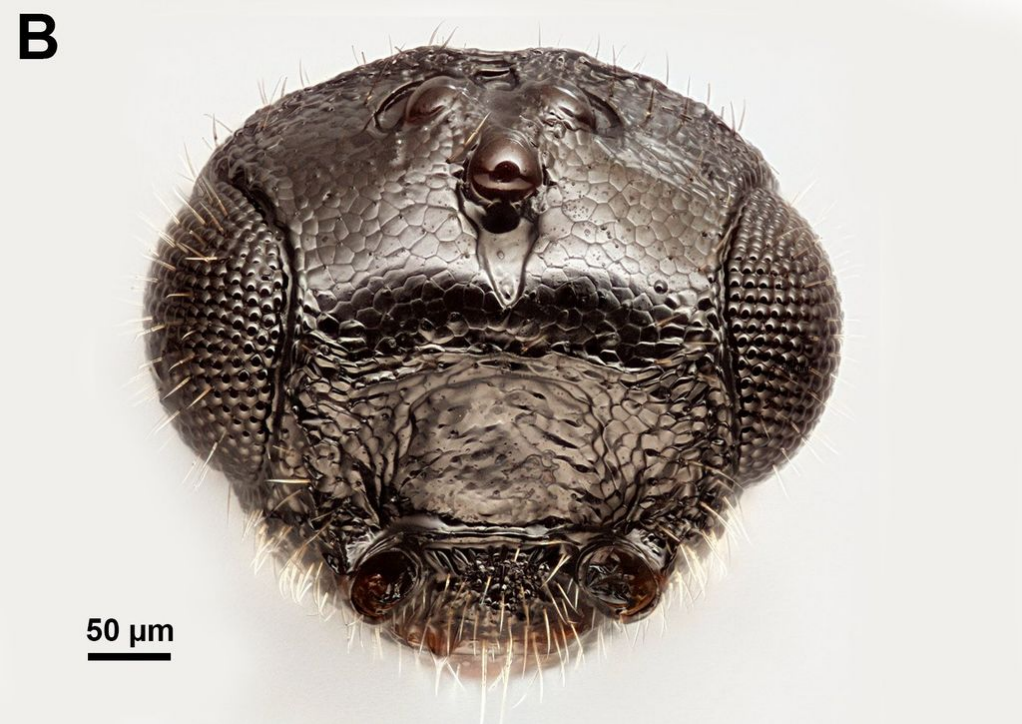
Head in anterior view.

**Figure 4a.**
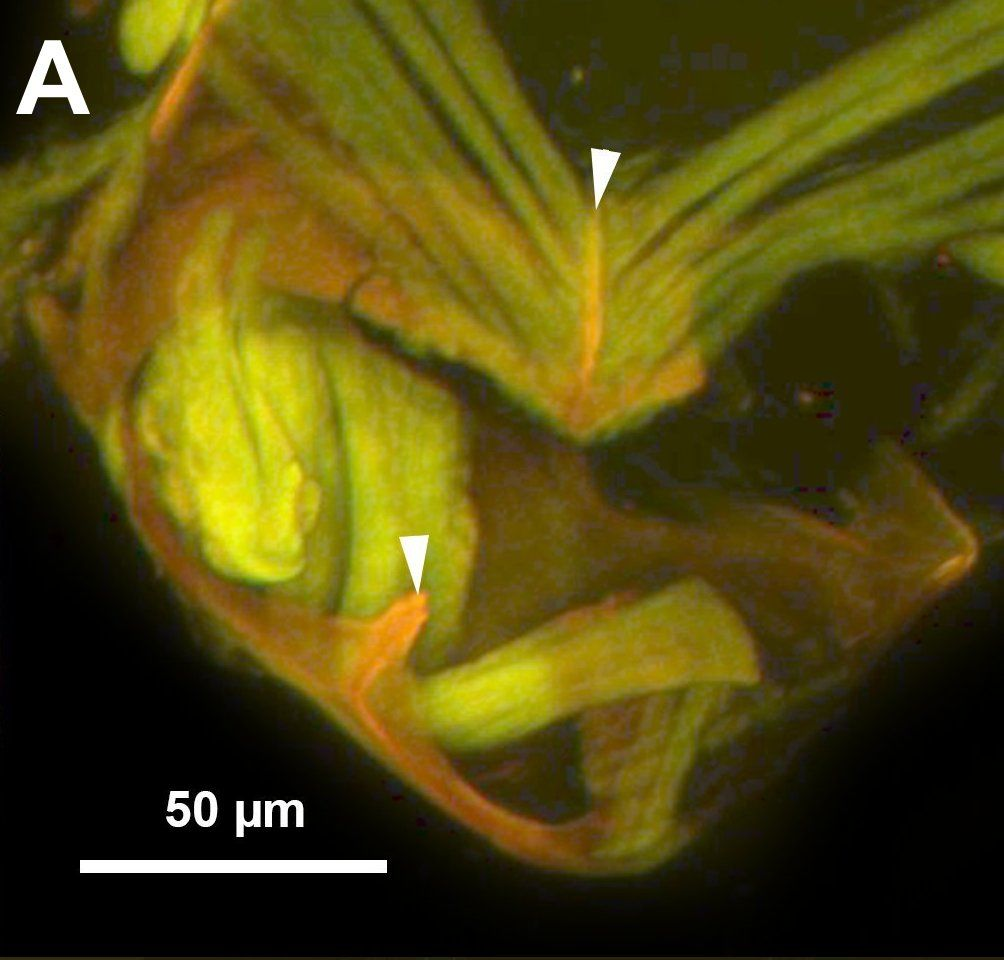
CLSM image showing the notch of cupula and muscle attachment apodeme of cupula.

**Figure 4b.**
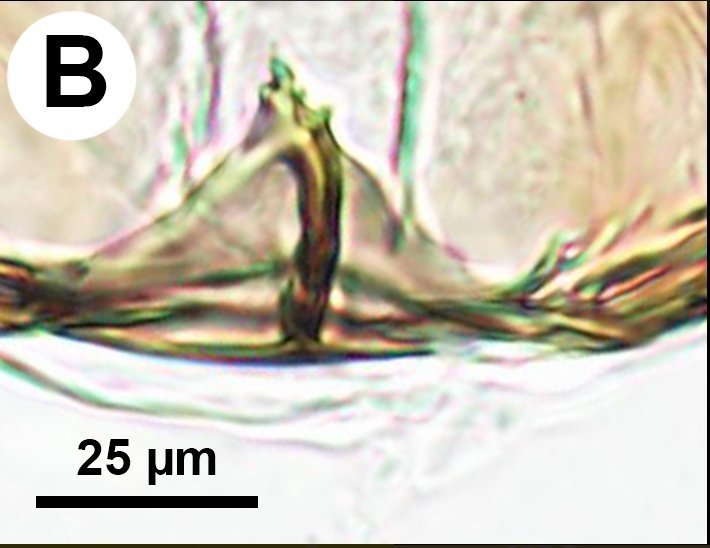
Brightfield image of cupula notch.

**Figure 4c.**
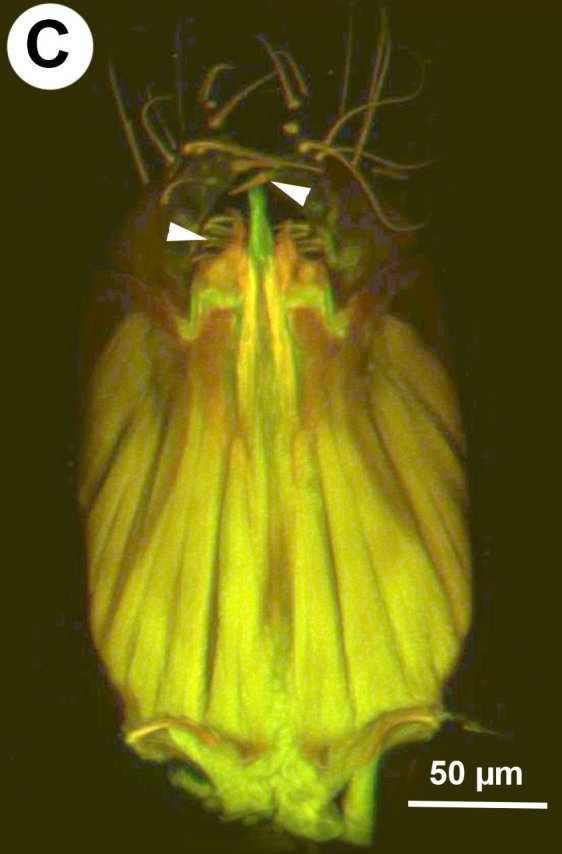
Male genitalia in ventral view, arrows indicate setal arrangement along proximal edge of harpe.

**Figure 4d.**
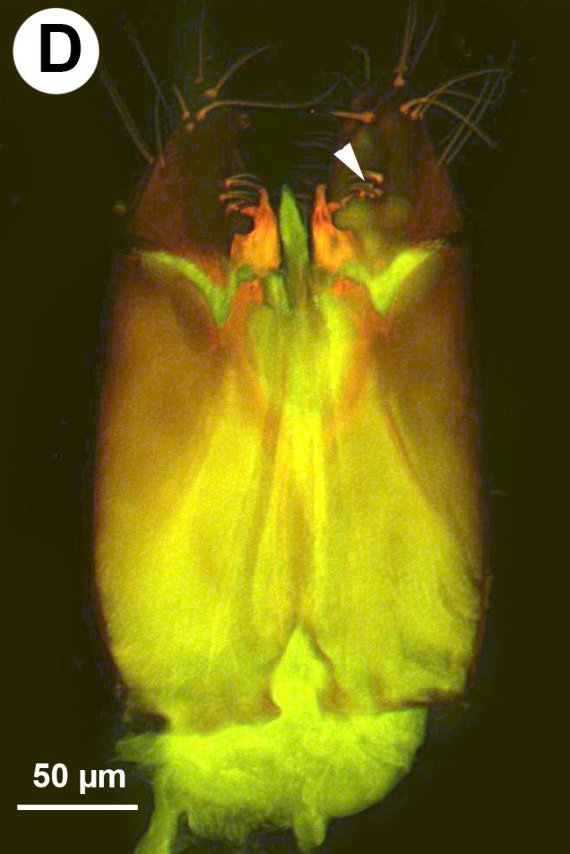
Male genitalia in dorsal view, arrows indicating setal patch of harpe.

**Figure 5a.**
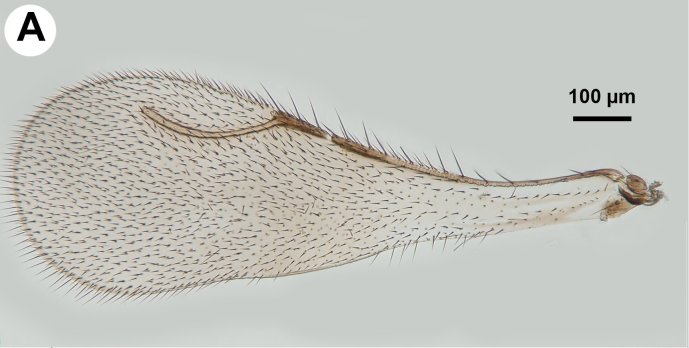
Forewing showing reduced pterostigma and stigmal margin of Ceraphronidae.

**Figure 5b.**
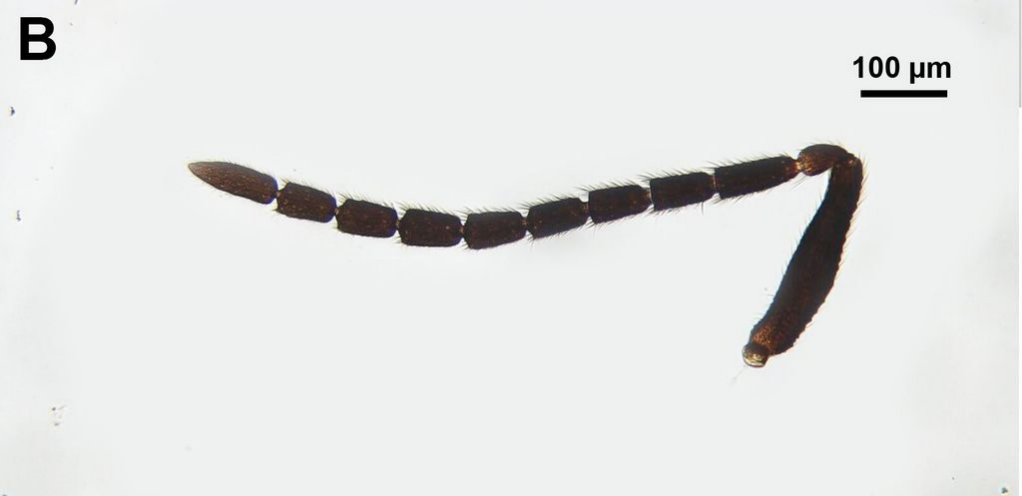
Antenna of male.
